# Compulsory Admissions of Patients with Mental Disorders in Europe: State of The Art on Ethical and Legislative Aspects

**DOI:** 10.1192/j.eurpsy.2022.197

**Published:** 2022-09-01

**Authors:** D. Wasserman

**Affiliations:** Karolinska Institutet, National Centre For Suicide Research And Prevention Of Mental Lll-health, Solna, Sweden

## Abstract

Globally, mental health legislation has been changing and evolving throughout time. Compulsory admission is when patients with mental disorders are admitted to a psychiatric unit against their own will to receive treatment. In Europe, as public attitudes and practices shifted, compulsory admission procedures were reformed to ensure patient rights and safety. Differences however exist for compulsory admission procedures between countries. In 2018, a survey was created and disseminated by the European Psychiatric Association Ethics Committee to National Psychiatric Associations within 40 European countries to learn more about the legislation, key actors involved, and reasons for admission. Results showed that approximately half of the responding countries required an independent medical expert, typically a psychiatrist, to be involved in the procedure. Most countries by law required the involvement of a court-appointed judge in making the decision about compulsory admission and to review the case after a certain period of time. All but one country have time limits on legal decisions for compulsory admission. Further, patients have the right to obtain legal counsel to assist them through the process of appeal or the decision in most countries. The primary reasons for admission were the patient being a danger to themselves or others. Despite continued efforts, stigma still surrounds mental health disorders. Public awareness and increased knowledge are needed to improve the perception of compulsory admissions. Moving forward, it is necessary to create educational courses, as well as written guidelines for key actors, to stimulate good practice and promote voluntary treatment (Wasserman et al. 2020; https://doi.org/10.1192/j.eurpsy.2020.79).

**Disclosure:**

No significant relationships.

